# Functional Evaluation of Embedded Modular Single-Branched Stent Graft: Application to Type B Aortic Dissection With Aberrant Right Subclavian Artery

**DOI:** 10.3389/fcvm.2022.869505

**Published:** 2022-05-02

**Authors:** Xuehuan Zhang, Duanduan Chen, Mingwei Wu, Huiwu Dong, Zhengdong Wan, Heyue Jia, Shichao Liang, Jun Shao, Jun Zheng, Shangdong Xu, Jiang Xiong, Wei Guo

**Affiliations:** ^1^School of Life Science, Beijing Institute of Technology, Beijing, China; ^2^School of Medical Technology, Beijing Institute of Technology, Beijing, China; ^3^Department of Vascular and Endovascular Surgery, The First Medical Center of People's Liberation Army of China, Beijing, China; ^4^Department of Ultrasound Diagnosis, The First Medical Center of People's Liberation Army of China, Beijing, China; ^5^Department of Vascular and Endovascular Surgery, The First Affiliated Hospital of Yangtze University, Jingzhou, China; ^6^Department of Cardiothoracic Surgery, The Second Affiliated Hospital of Nanjing Medical University, Nanjing, China; ^7^Center of Cardiac Surgery, Beijing Anzhen Hospital, Beijing Institute of Heart, Lung and Vascular Diseases, Capital Medical University Beijing Aortic Disease Center, Beijing, China; ^8^Department of Vascular and Endovascular Surgery, Hainan Hospital, People's Liberation Army of China, Hainan, China

**Keywords:** functional evaluation, aortic dissection, aberrant right subclavian artery, embedded modular single-branched stent graft, hemodynamics, aortic remodeling

## Abstract

**Background:**

Endovascular repair of type B aortic dissection (TBAD) with aberrant right subclavian artery (ARSA) is challenging due to anatomical complexity. The embedded modular single-branched stent graft (EMSBSG) could solve this problem. However, the hemodynamic efficacy of this innovative technique has not been fully assessed. This study aimed to propose morphometric and functional indicators to quantify the outcomes of EMSBSG in treating TBAD with ARSA.

**Material and Methods:**

A patient who had TBAD with ARSA underwent EMSBSG implantation was admitted. Computational fluid dynamics (CFD) and three-dimensional structural analyses were conducted based on CTA datasets before the operation (Pre-1) and at 4 and 25 days after EMSBSG implantation (Post-1 and Post-2). Quantitative and qualitative functional analyses were conducted via pressure-, velocity- and wall shear stress (WSS) -based parameters, such as the luminal pressure difference (LPD), total energy loss, and flow distribution ratio. By precisely registering the aortas at the three time points, parameter variations in the EMSBSG region were also computed to investigate the prognostic improvement after EMSBSG implantation.

**Results:**

The first balance point of LPD distally shifted to the abdominal aorta in Post-1 by a distance of 20.172 cm, and shifted out of the dissected region in Post-2, indicating positive pressure recovery post EMSBSG. The flow distribution ratios of all aortic arch branches increased after EMSBSG implantation. A positive normal deformation index in the EMSBSG region confirmed true lumen expansion; dominant AR_N_ (area ratio of negative value) of pressure and WSS-based parameters indicated an improved prognosis from Post-1 to Post-2.

**Conclusions:**

The short-term results of EMSBSG in treating TBAD with ARSA proved to be promising, especially in EMSBSG region. Comprehensive evaluation could provide new insight into the therapy of TBAD with ARSA. Thus, it might guide the further management of complex aortic arch lesions.

## Introduction

Aberrant right subclavian artery (ARSA), originating from the proximal portion of the descending thoracic aorta, is a common variant of the aortic arch (1). However, the association of type B aortic dissection (TBAD) and ARSA is rarely encountered. It has been reported that the acute angle of the ARSA weakens the aortic wall (2), leading to the formation of the primary tear near the descending aortic isthmus and ARSA (3). The treatment procedure is limited due to the location of the primary tear and ARSA.

To date, there is no consensus on the treatment of TBAD associated with ARSA. Conventional open surgery is the most common option for TBAD with ARSA (2, 4). However, complex open surgical procedures may be associated with relatively high perioperative mortality and morbidity (5). New endovascular treatment techniques including chimney, periscope and fenestration have been applied to treat this pathology (3, 6, 7). However, chimney technique has been reported to be associated with high endoleak and reintervention rates (8) and the safety of fenestration (*in situ* or *in vitro*) remains unclear.

The branched stent graft technique might be an alternative for aortic arch pathologies. The embedded modular single-branched stent graft (EMSBSG) is an endovascular device specially designed for the lesions involving the branch vessel. EMSBSG could not only restore the aortic morphology but also preserve the blood flow for the branches. The high flexibility of this EMSBSG technique might also contribute to avoiding potential device-related complications. This technique could adapt to the patient-specific characteristics *e.g*. the branch vessels with particular angles, thus facilitating the safety of managing lesions that involve the branch vessels, such as TBAD with ARSA.

However, the efficacy of EMSBSG in treating TBAD with ARSA has not been fully assessed. Herein we retrospectively reported a patient who had TBAD with ARSA and treated successfully with EMSBSG technique. CFD was used to evaluate the hemodynamic features before and after EMSBSG treatment. Detailed morphometric and functional information might serve as a reliable reference for assessing therapeutic effect of EMSBSG in treating TBAD with ARSA, thus guiding future clinical therapy in managing the variant anatomy of the aortic arch.

## Materials and Methods

### Patient and EMSBSG Device

**Figure 1:**
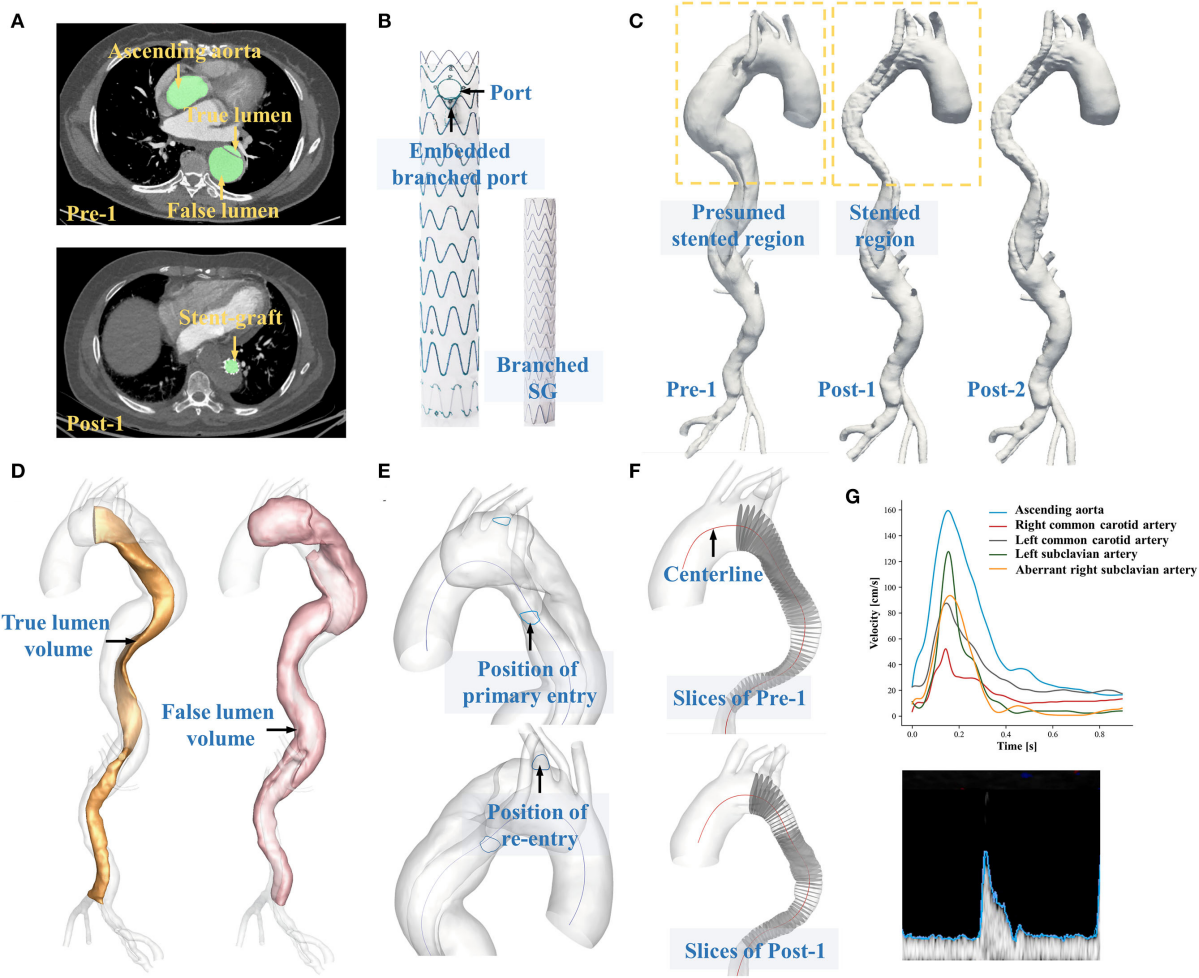
**(A)** Displays the segmentation of CTA datasets. **(B)** Shows the structural components of EMSBSG system. **(C)** Displays the 3D reconstructed preoperative and postoperative models. **(D)** Shows the separation of true lumen and false lumen. **(E)** Illustrates the position of tears. **(F)** Displays the extraction of a series of perpendicular slices along the TL centerline. **(G)** Displays the extraction of boundary conditions via Doppler ultrasound data.

A 59-year-old female with the history of hypertension who was diagnosed with chronic TBAD with ARSA in 2015 was admitted. The ARSA arose from the proximal portion of the descending thoracic aorta. After 6 years of optimal medical treatment, she was readmitted and operated on because of 1-month intermittent dull chest pain. The primary entry was located near the ARSA. A re-entry tear was detected at the beginning of the left subclavian artery (LSA) (Figure 1E). The patient refused open surgery or hybrid repair. Therefore, EMSBSG, specifically designed for aortic arch lesion, was used to seal the tears and hence restore the aortic morphology. The EMSBSG system was manufactured by Hangzhou Endonom Medtech Co., Ltd (Hangzhou, China) according to individual needs. In the current study, the EMSBSG system is comprised of the main aortic stent graft (34 ^*^ 26 ^*^ 160 mm) with an embedded branch port for ARSA, the stented port for LSA, and the branched stent graft, as shown in Figure 1B. Detailed information on the steps to perform the endovascular repair procedure using EMSBSG system is presented in the Supplementary Data Sheet 1. The EMSBSG system is in the clinical trial, and full details of the clinical studies are available at ClinicalTrials.gov (NCT04765605). This study was approved by the Institutional Review Board of the Chinese PLA General Hospital (S2020-010-01). Written informed consent was provided by this patient prior to this study.

### Image Acquisition and Geometry Reconstruction

The patient underwent three CTA scans before the operation (Pre-1) and at 4 and 25 days after EMSBSG implantation (Post-1 and Post-2). All three CTA datasets were acquired via a dual-source CT scanner (Brilliance iCT256, Royal Philips, Dutch). Detailed scanning parameters are available in Supplementary Data Sheet 2. DICOM files of Pre-1, Post-1 and Post-2 CTA datasets were exported with slice numbers of 614, 1,219 and 641, respectively. Image segmentations and three-dimensional vessel configuration reconstructions were conducted via the automatic image processing module developed by our team, with the segmentation time of 14.19 ± 2.15 s for each case (9), as shown in Figure 1A. The image segmentations were subsequently reviewed for each slice by an expert with rich experience in reading CT images with supervision by one professional vascular surgeon. Manual correction was performed as necessary. The luminal region with patency was segmented, and the cross-sectional contours of reconstructed geometries were mapped back to CTA images to ensure that the 3D-reconstructed models presented the actual outline of the vessel lumen. For each patient, segmentations were performed from the ascending aorta to the distal end of the dissection. The reconstructed models were finally exported in triangulation mesh (STL format) to facilitate the calculation of any morphological parameters. Figure 1C shows the 3D-reconstructed aortic models of Pre-1, Post-1 and Post-2.

### 3D Morphological Measurements

Morphological parameters were measured on the basis of 3D-reconstructed models. To quantify the geometric variations of different models, an optimal alignment of the Pre-1, Post-1 and Post-2 models is a prerequisite procedure. The iterative closest point algorithm, which is the most popular method for 3D rigid registration (10), was used to transform postoperative models to properly align with the preoperative model. The algorithm was developed via the Visualization Toolkit (VTK) package in Python 3.9 (https://vtk.org).

When the preoperative and postoperative models were aligned, luminal volume changes for the true lumen (TL) and false lumen (FL) were calculated (Figure 1D) and centerlines were also extracted. To further investigate the efficacy of EMSBSG insertion, the morphological changes of the main aortic grafting region (MAGR) were also computed including volume changes and the parameters along the centerline of MAGR. In detail, a series of slices perpendicular to the centerline with an interval of 1.0 mm were first extracted (Figure 1F) and then the morphological characteristics of each slice were computed including the area, circumference, equivalent diameter, transverse diameter, longitudinal diameter and the ratio between the transverse and longitudinal diameters (aspect ratio). The curvature and tortuosity of MAGR were also computed on the basis of the centerline. Tortuosity was defined as equation 1 (Eq. 1), where *d* indicates the linear distance of the centerline and *l* indicates the distance along the centerline. The methods used to measure each parameter are further described in Supplementary Data Sheet 3. The normal deformation index of MAGR from Post-1 to Post-2 was calculated to quantify luminal expansion over time.


(1) $$\begin{array}{r} Tortuosity\;=\;1\;-\;\frac{d}{l} \end{array}$$

### Doppler Ultrasound and Boundary Conditions

Time-variant velocities at the ascending aorta, right common carotid artery, left common carotid artery, LSA and ARSA were measured via Doppler ultrasound of the patients. At each measurement site, an appropriate ultrasound probe was employed (Supplementary Table 1; Supplementary Data Sheet 4). The upper edge of the velocity sonogram was extracted as the variation in the maximum velocity at the measured site (Figure 1G). The measurements were used to provide patient-specific velocity boundary conditions for the computational model. In the current study, Doppler ultrasound data were acquired before treatment and at 4 days after treatment. Therefore, Post-1 and Post-2 were given the same boundary conditions. Supplementary Figure 2 shows the velocity boundary conditions for Pre-1, Post-1 and Post-2 (Supplementary Data Sheet 4). The flow distribution ratio for each aortic arch branch, including the right common carotid artery, left common carotid artery, LSA and ARSA was computed on the basis of the Doppler ultrasound data, which was defined as the ratio of the branching flow to the inflow. Details of pulsatile waveforms of pressure at the celiac artery, superior mesenteric artery, renal arteries and outlets at iliac arteries were obtained from a previous study and used as pressure boundaries (11). In our previous study (12), measured pressure was compared with the data we used in this study, confirming the rationality of pressure boundary conditions.

### Numerical Models

3D aortic models were meshed using ICEM (Ansys Inc., Canonsburg, USA) with tetrahedral elements in the core region and prismatic cells (10 layers) in the boundary layers near the aortic wall. The grid resolutions for Pre-1, Post-1 and Post-2 were 3397577, 2211420 and 2329161 cells, respectively. The values of the cardiac cycle extracted from Doppler ultrasound examination were 55 and 85 beats/min for pre- and post-treatment data, respectively. Temporal discretization of numerical models was assigned to be 50 steps per cycle. The transport equations of time-dependent flow were numerically solved using a finite volume solver, CFD-ACE (ESI Group, Paris, France) (Supplementary Data sheet 5). The blood was assumed as Newtonian and incompressible with a density of 1,044 kg/m^3^ and dynamic viscosity of 0.00365 kg m^−1^ s^−1^. For simulation purposes, the no-slip and rigid arterial wall was ascertained. For each model, four cardiac cycle simulations were carried out to obtain a periodic solution, and the results of the final cycle were presented for postprocessing and analysis. Grid and time step independence tests were conducted in our previous study (13, 14), confirming the rationality of the base grids and base temporal resolution in this study.

### Analysis of Hemodynamic Parameters

Qualitative and quantitative analyses were used to investigate the hemodynamic patterns at Pre-1, Post-1 and Post-2. The aortic pressure distribution and flow patterns at the systolic peak were studied. Wall shear stress (WSS) related parameters were selected to analyze the impact of EMSBSG insertion, including time-averaged wall shear stress (TAWSS), oscillatory shear index (OSI) and relative residence time (RRT). Details of the computation of WSS-related parameters are presented in Supplementary Data Sheet 6.

The luminal pressure difference (LPD) and the first balance position (FBP) were computed, which have been proven to be an efficient functional indicator of TBAD in our previous study (15). The spatial variation of LPD along the aorta and shift of the FBP between Pre-1, Post-1 and Post-2 were assessed. The total energy loss was evaluated in this study. The energy difference between the inlet and outlets of the aorta indicated the total energy loss during a cardiac cycle (16), which was defined as follows.


(2) $$\begin{array}{r} EL=\sum_{Inlet}\left(TP{\;}^{*}\;Q\right)-\sum_{Outlets}\;\left(TP{\;}^{*}\;Q\right) \end{array}$$

Where Q is the blood flow rate. TP indicates the total pressure.


(3) $$\begin{array}{r} TP=\frac{1}{2}\rho |\vec{u}{|}^{2}+P \end{array}$$

Where *u* and P refer to velocity and pressure, respectively.

The alterations in systolic peak pressure, TAWSS, OSI and RRT from Post-1 to Post-2 of MAGR were computed. In the current study, Δ was used to denote the difference of the abovementioned parameter between Post-1 and Post-2, which was defined as Eq. 4.


(4) $$\begin{array}{r} \Delta X=V_{Post-1}^{X}-V_{Post-2}^{X} \end{array}$$

Where *X* is the previously mentioned parameters. Area ratios (AR) with positive value (AR_P_) and negative value (AR_N_) were subsequently computed for each Δ*X*, which was defined as Eq. 5.


(5) $$\begin{array}{l} AR_{P}=\frac{AR_{P}}{AR_{P}+AR_{N}}\\ AR_{N}=\frac{AR_{N}}{AR_{P}+AR_{N}} \end{array}$$

To capture different aspects of the local hemodynamics, parameters including systolic peak pressure, TAWSS, OSI and RRT were averaged across the circumferences orthogonal to the centerline of MAGR and ARSA (every 1 mm).

## Results

### Morphometric Analysis

**Figure 2:**
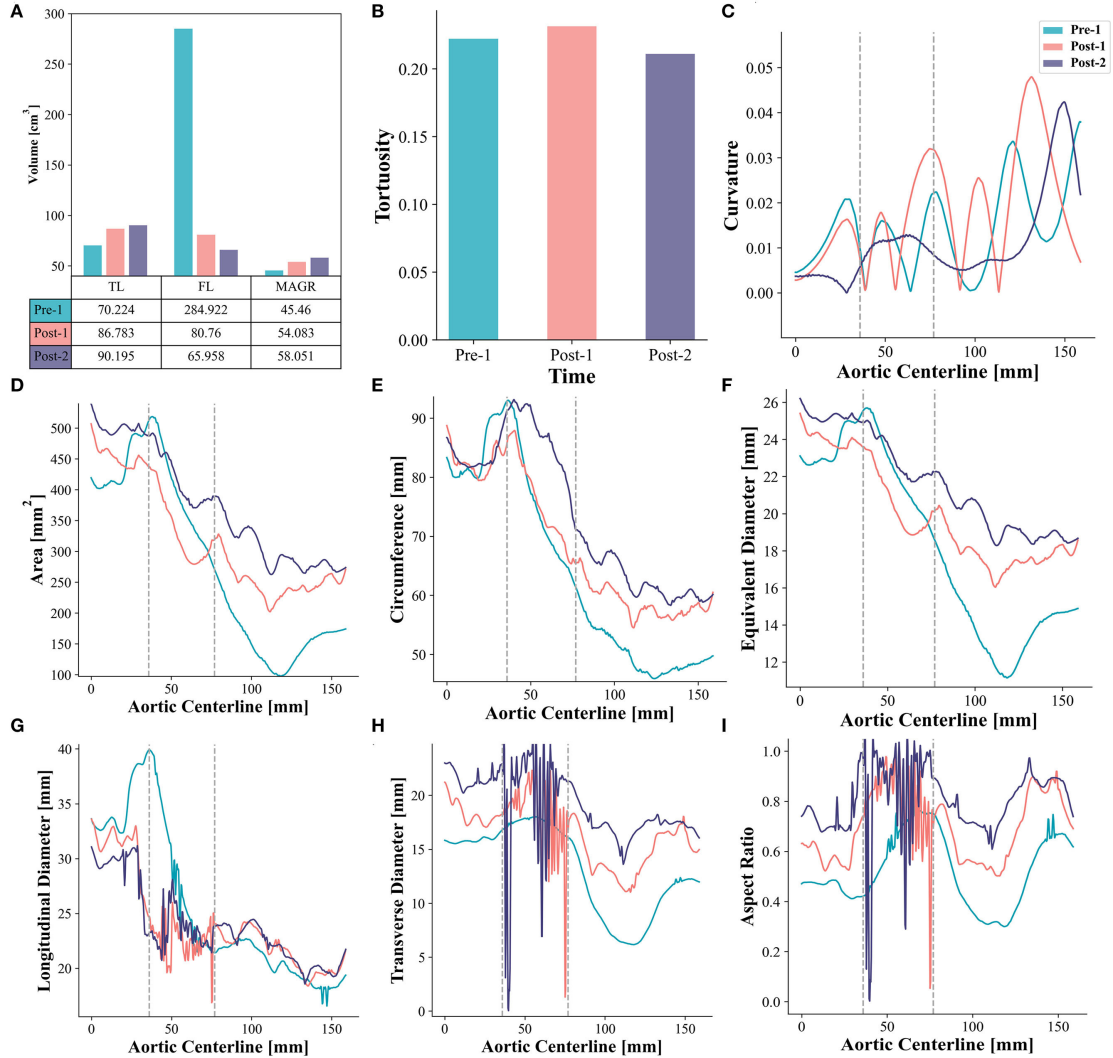
Morphometric analysis **(A)** Illustrates the volume changes. **(B)** Shows the tortuosity changes. **(C)** Displays curvature alterations. **(D–F)** Show the alterations of area, circumference, and equivalent diameter, respectively. **(G–I)** Display the result of longitudinal diameter, transverse diameter and aspect ratio for Pre-1, Post-1 and Post-2, respectively. The region between two gray dotted lines indicates the embedded branch port zone. TL, true lumen; FL, false lumen; MAGR, main aortic grafting region.

Morphological characteristics before and after EMSBSG insertion were quantified. As shown in Figure 2A, both the volume of TL and MAGR increased, while the volume of FL decreased. A slight increase in tortuosity was detected from Pre-1 to Post-1 with values of 0.222 and 0.231, respectively. However, the tortuosity reduced to 0.211 in Post-2, which was smaller than that in Pre-1. Furthermore, Figure 2C indicates the fluctuations of the curvature. The results showed that the curvature of Pre-1 and Post-1 is not only larger than that of Post-2 but also changes more dramatically than that of Post-2. The area, circumference and equivalent diameter along the MAGR centerline showed an overall increase except a slight reduction in the embedded branch port at Post-1. The region of the embedded branch port is indicated between two dotted lines in Figure 2; while Post-2 showed luminal expansion compared with Post-1. The results of longitudinal diameter, transverse diameter and aspect ratio showed that the EMSBSG system could enlarge the vessel lumen. Both Post-1 and Post-2 showed an increase in transverse diameter and aspect ratio, and Post-2 was larger than Post-1, as shown in Figures 2H,I. The tear information for this patient at different time-points were concluded in Supplementary Table 2; Supplementary Data Sheet7.

### Pressure Fields and Velocity

**Figure 3:**
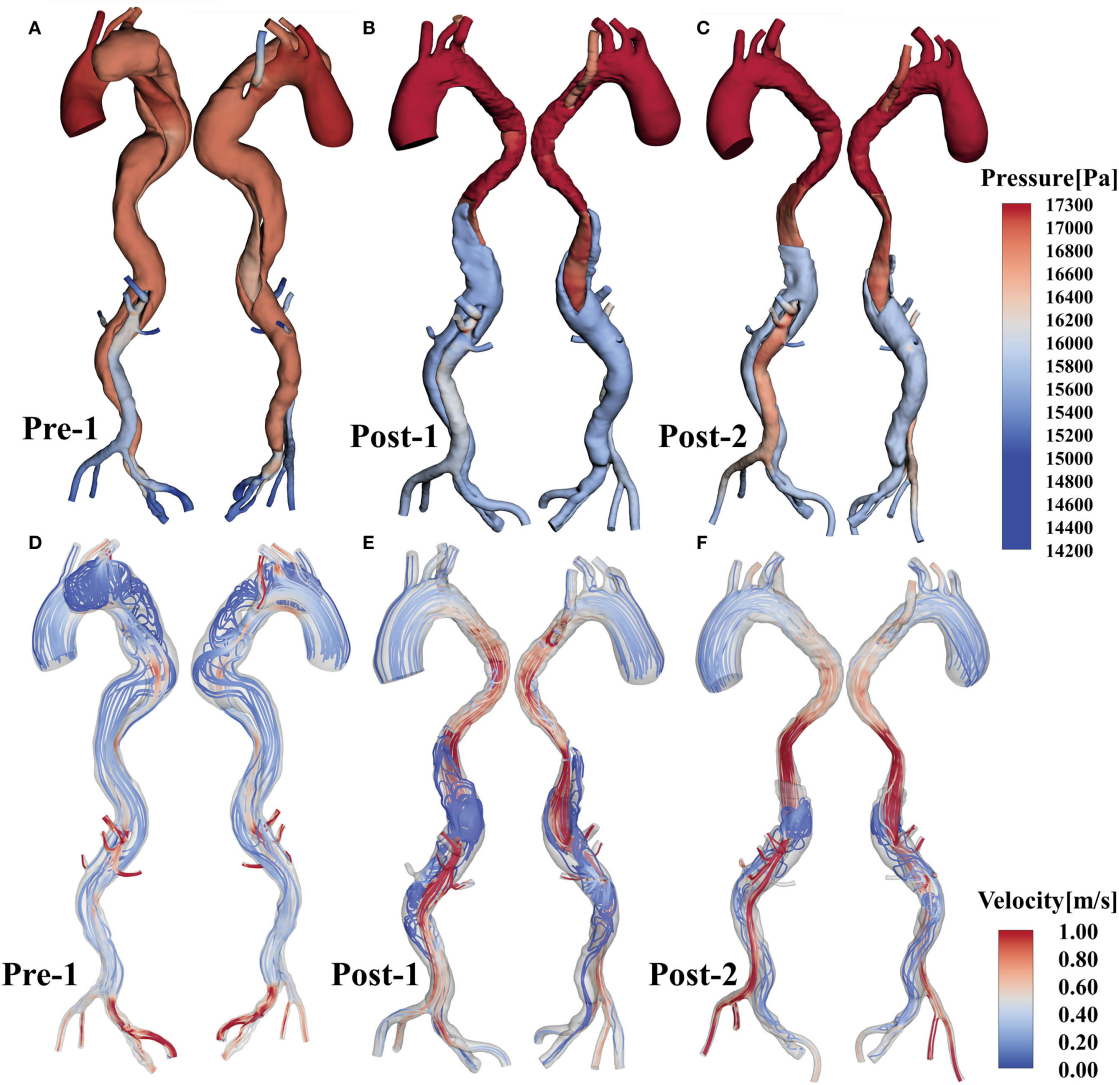
**(A–C)** Show the pressure distributions of Pre-1, Post-1 and Post-2, respectively. **(D–F)** Display flow patterns at peak systole for Pre-1, Post-1 and Post-2, respectively.

Pressure distributions and velocity streamlines for Pre-1, Post-1 and Post-2 at the systolic peak are shown in Figure 3, where the color map of pressure and velocity magnitude were restricted to a certain range for all cases to assist visualization. Post-1 showed higher pressure than Pre-1. The pressure dropped at the distal region near the EMSBSG region from Post-1 to Post-2. However, the pressure increased in both TL and FL in the abdominal region. The ARSA grafting region showed a continuous pressure rise after treatment. At the systolic peak, fast and organized flow was found in TL; while vortical and relatively slow flow was present in FL. The flow accelerated in MAGR of Post-1, while delayed in Post-2. In the ARSA grafting region, the velocity showed a reduction from Pre-1 to Post-1, while high-speed flow was detected in the root of ARSA in Post-1. In Post-2, the flow has a significant slow-down.

### WSS-Based Parameter Analysis

**Figure 4:**
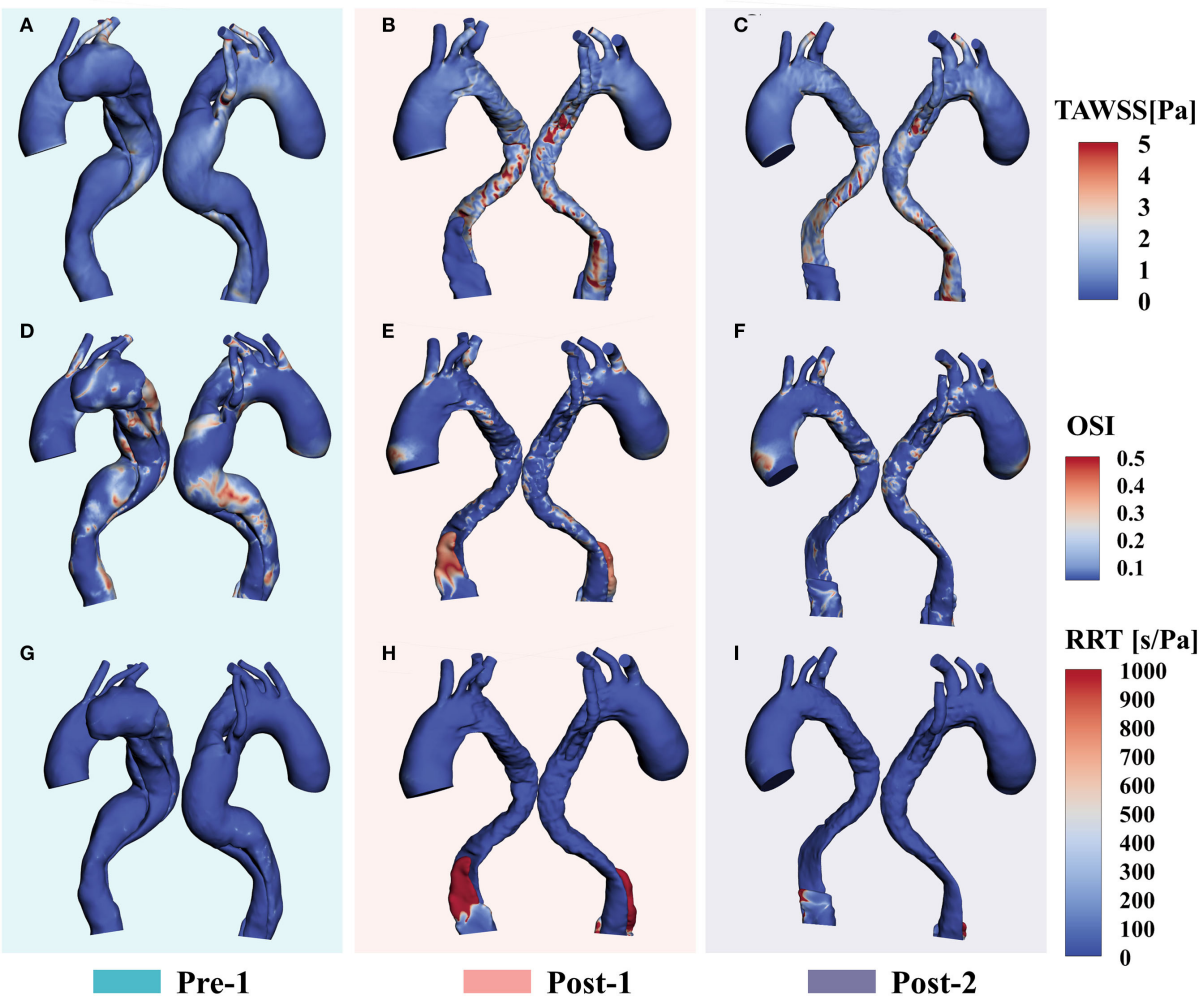
WSS-based parameter **(A–C)** Show the TAWSS distributions. **(D–F)** Display the results of OSI contour plots. **(G–I)** Illustrate the RRT patterns. WSS, wall shear stress; TAWSS, time-averaged wall shear stress; OSI, oscillatory shear index; RRT, relative residence time.

Loading patterns are crucial for hemodynamic analysis of vessel remodeling after stent-graft implantation. Figure 4 shows the distributions of TAWSS, OSI and RRT of Pre-1, Post-1 and Post-2. Before treatment, we observed a high TAWSS concentration in the root of ARSA (Figure 4A). With EMSBSG insertion, TAWSS elevated in the both MAGR and ARSA grafting regions at Post-1 while declined at Post-2, as shown in Figures 4B,C. OSI contour plots are shown in Figures 4D–F. There were some high OSI regions before surgery, while OSI decreased in the EMSBSG region at Post-1, and Post-2 showed a further reduction. Figure 4G displays the low RRT of the preoperative model, while high RRT appeared in the FL region of Post-1, as indicated in Figure 4H. The zone showed high RRT in Post-1 thrombosed in Post-2, as shown in Figure 4I.

### Quantitative Analysis of Hemodynamic Status

**Figure 5:**
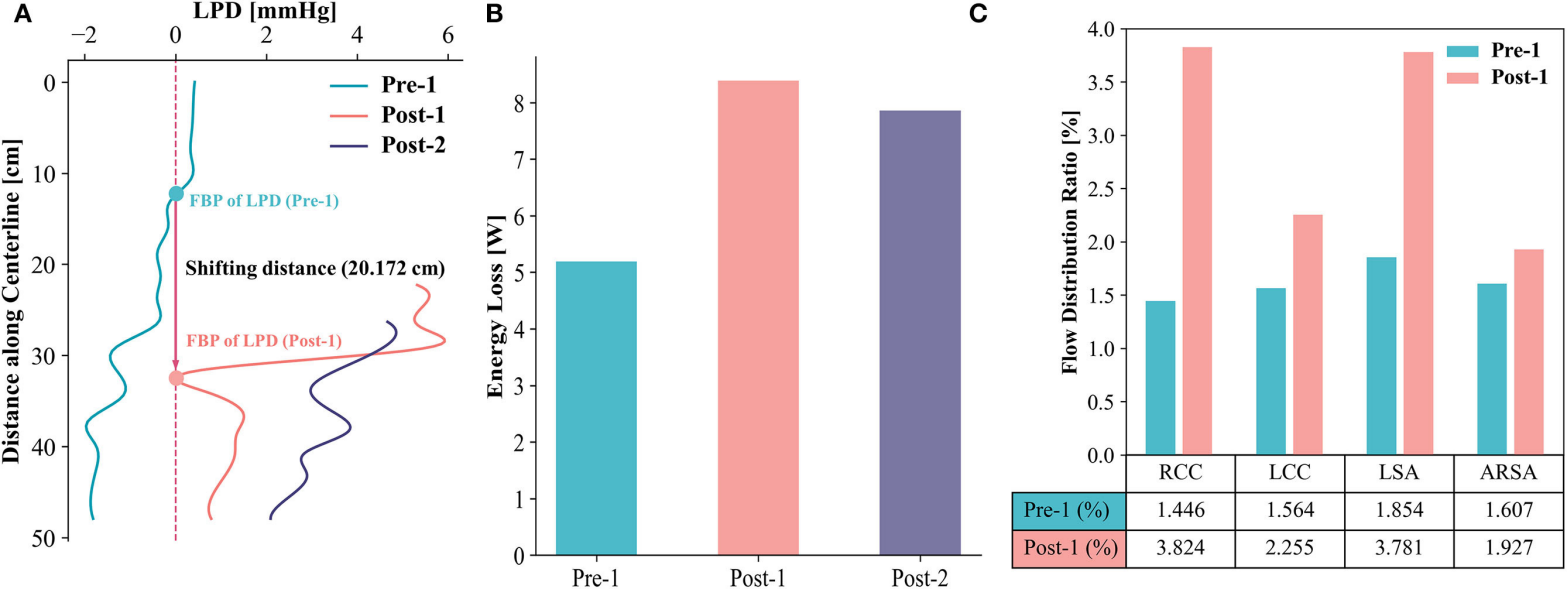
**(A)** Shows the time-averaged LPD curves of Pre-1, Post-1 and Post-2. The FBP shifting distance is 20.172 cm after intervention. **(B)** Displays the total energy loss of Pre-1, Post-1 and Post-2. **(C)** Indicates the changes of flow distribution ratio from Pre-1 to Post-1. LPD, luminal pressure difference; FBP, first balance position; RCC, right common carotid artery; LCC, left common carotid artery; LSA, left subclavian artery; ARSA, aberrant right subclavian artery.

To investigate the LPD between the TL and FL, a series of slices perpendicular to the centerline of the TL were extracted and the net pressures in the TL (P_TL_) and FL (P_FL_) on each slice over a cardiac cycle were calculated. Figure 5A displays the LPD (LPD = P_TL_ – P_FL_) for each case. The FBP of LPD shifted to the abdominal aorta distally in Post-1, with a shift distance of 20.172 cm from Pre-1 to Post-1. Moreover, the FBP of the LPD shifted out of the dissected region at Post-2. The energy loss was compared among Pre-1, Post-1 and Post-2 (Figure 5B). Pre-1 showed the lowest energy loss with a value of 5.19 W and it increased to 8.39 W after EMSBSG procedure. However, a slight drop was observed in Post-2 at a value of 7.86 W. The flow distribution ratio showed a general increase for each aortic arch branch after EMSBSG intervention, as shown in Figure 5C.

**Figure 6:**
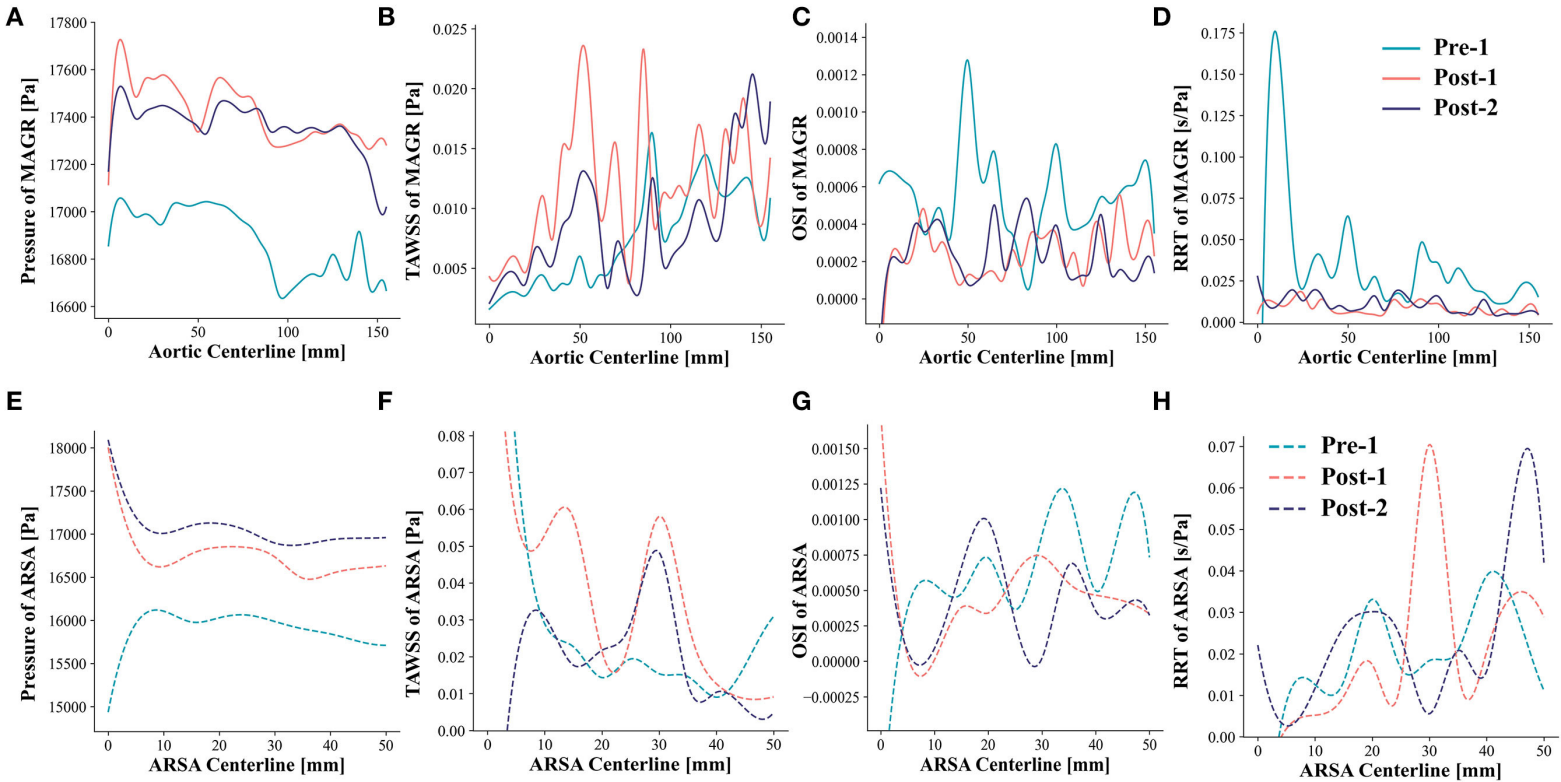
Patterns of pressure, TAWSS, OSI and RRT along the MAGR and ARSA region. **(A–D)** Show the hemodynamic patterns for MAGR via solid lines. **(E–H)** Demonstrate the hemodynamic patterns for ARSA region using dotted lines. MAGR, main aortic grafting region; ARSA, aberrant right subclavian artery; TAWSS, time-averaged wall shear stress; OSI, oscillatory shear index; RRT, relative residence time.

Examinations of the spatial differences in peak systolic pressure, TAWSS, OSI and RRT demonstrated the patterns along the MAGR and ARSA regions (Figure 6). In MAGR, pressure and TAWSS increased after EMSBSG placement, while declined slightly at Post-2 (Figures 6A,B). Both OSI and RRT showed a significant decrease from Pre-1 to Post-1 (Figure 6C). In the ARSA grafting region, the peak systolic pressure showed a continuous rise after EMSBSG implantation, as shown in Figure 6E. TAWSS and RRT increased first at Post-1 and then declined at Post-2 (Figures 6F,H). The hemodynamic parameter alterations from Post-1 to Post-2 were further analyzed (detailed later).

### Prognostic Improvement Analysis

**Figure 7:**
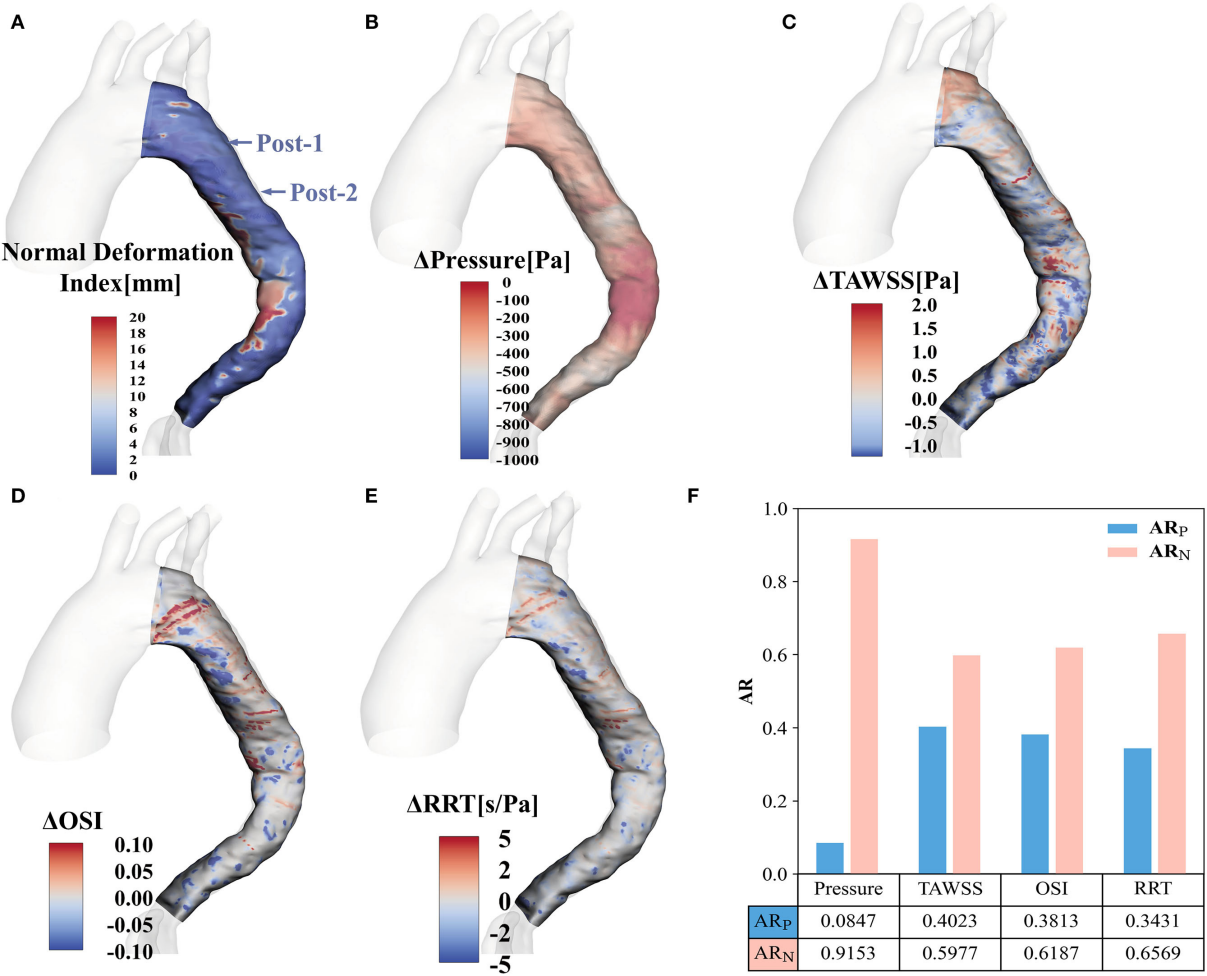
Contour plots for the evolution fields by registration **(A)** Shows the normal deformation index field. **(B–E)** Display the alterations of pressure, TAWSS, OSI and RRT, respectively. **(F)** Illustrates the AR of positive value and negative value for pressure, TAWSS, OSI and RRT. TAWSS, time-averaged wall shear stress; OSI, oscillatory shear index; RRT, relative residence time; ARP, area ratio with positive value; ARN, area ratio with negative value.

To quantify the prognostic improvement, the evolution fields obtained by registration are shown in Figure 7. Figure 7A displays the normal deformation index contour plot, which illustrates the vessel lumen changes over time after EMSBSG placement. Figures 7B–E show the alterations of systolic peak pressure, TAWSS, OSI and RRT from Post-1 to Post-2, respectively. Figure 7F displays the AR of each previously mentioned parameter. The AR_N_ was larger than the AR_P_ for all factors, indicating that all of these parameters decreased from Post-1 to Post-2, which might designate a good prognostic result in MAGR.

## Discussion

This study is based on a longitudinal dataset of one patient with originally TBAD accompanied by ARSA, treated with EMSBSG. The EMSBSG technique used to treat TBAD with ARSA should exclude the principal tear, restore TL morphology and preserve the blood flow toward the arch branch vessels. To investigate the therapeutic effect of this novel technique, 2 key factors for the vascular system including morphology and hemodynamics were computed. The former, representing the structural features of vessels, is now widely used clinically. The latter, as a functional estimation, is related to pathogenetic mechanisms and might contribute to prognosis prediction after management.

CFD is widely used in TBAD to assess hemodynamic features, including flow patterns (13, 17), post-stenting flow effects (15), thrombosis establishment (13, 18) and implantation plans (19, 20). These studies contributed greatly to our understanding of TBAD and its prognosis after treatment. However, the evaluation of EMSBSG in treating TBAD with ARSA has not been fully assessed. In the present study, patient-specific models were reconstructed based on CTA images and patient-specific flow boundaries were obtained from ultrasound velocimetry. A possible relationship between prognosis and morphometric and hemodynamic indicators was subsequently detected in this patient.

The results of volume analysis and morphology parameter changes along the MAGR centerline indicated TL and FL progression after EMSBSG implantation. The tortuosity increased in Post-1, while decreased in Post-2. This might be induced by the placement of the stent graft. In a short follow-up period, the stent complies with the aorta morphology, thus we could see an increase in tortuosity in Post-1. However, the stent graft has an inherent tendency to spring back to its initial straight status when passively bent at the aortic arch (21). Therefore, tortuosity decreased in Post-2. This case should be closely monitored in the future to avoid the stent-graft induced injury because the spring-back could generate stress on the greater curve at the distal end (21).

The SG implantation procedure might stiff the thoracic aorta and reduce the radial strain within the stented segment. Increased conduit stiffness elevates pulse wave velocity, leading to increased pulse pressure (22–24), thus resulting in adverse cardiac remodeling (25, 26). Moreover, flow acceleration could potentially indicate the risk of stent graft migration (27, 28). This information informed us to monitor high flow and pressure patterns after EMSBSG treatment. In the current study both pressure distributions and flow patterns increased at Post-1, while decreased at Post-2 in MAGR. Quantitative analysis of the AR of Δpressure also showed that AR_N_ was larger than AR_P_ (Figure 7F). The aortic lumen expanded significantly at Post-2 compared to Post-1. In fact, the flow environment is highly related to the morphology of the vessel. With the positive aortic modeling due to the stent expansion, the blood velocity and the pressure are commonly reduced. Our findings also indicated that the risk of stent migration was relatively low in our patient treated by EMSBSG. However, the velocity and pressure increased from the arch to the descending aorta in both Post-1 and Post-2. This might be induced by the embedded branch port of ARSA. Even though a reduction of velocity and pressure was obvious from Post-1 to Post-2, more follow-up images will be needed in the future to further evaluate the luminal remodeling. To further explore the pressure and flow patterns of other stent techniques, two cases with provisional extension to induce complete attachment technique (with bare mental stent) and traditional thoracic endovascular aortic repair technique (without bare mental stent) were also investigated, as shown in the Supplementary Figure 3; Supplementary Data Sheet 8.

Analyses of EMSBSG regional mechanisms were conducted to investigate the prognostic improvement. The normal deformation index in the whole MAGR quantifies the TL expansion from Post-1 to Post-2. The elevation in TAWSS and OSI could indicate poor prognosis, such as graft immigration (19). The dominant AR_N_ of TAWSS and OSI was observed in this study, suggesting a favorable prognosis in the EMSBSG region. High RRT has been found to correlate with thrombosis absorption (13, 29), which is usually considered a positive event in TBAD management. In our patient, high RRT appeared at the FL proximal tip, where thrombosed at Post-2 (Figures 4H,I), consistent with our previous findings (13). However, as shown in Supplementary Figure 4; Supplementary Data Sheet 9, low RRT in abdominal FL may imply the negative remodeling of FL; high TAWSS regions were also observed in this region, which might not lead to constructive FL remodeling (18, 30). Low RRT and high TAWSS in the abdominal TL region may be induced by re-entry tears located above the celiac artery and those near the right renal artery (Supplementary Figure 5; Supplementary Data Sheet 9). Therefore, frequent monitoring of the abdominal aorta region in this patient is needed in the future.

FBP shifted out of the dissected region at Post-2 (25 days after intervention), implying positive aortic remodeling (15). An instant rise was observed in total energy loss from Pre-1 to Post-1 while a subsequent decline appeared in Post-2. This potentially suggests that although the resistance of the EMSBSG region increases in a short period, it will decrease with dilation of the arterial wall. The ability of EMSBSG to preserve the blood flow for branched vessels was examined via computation of the flow distribution ratio. As shown in Figure 5C, we could see elevated flow distribution ratios for each aortic arch branch after the treatment procedure. The results showed that EMSBSG could maintain the flow toward the branch vessels, although the flow distribution ratios of branches were still smaller than that of the normal model (31), which might be due to the short follow-up period (4 days after intervention). Furthermore, the existence of dissection and the reverse tear located near the LSA may account for the limited blood flow into branches in Pre-1.

This study also investigated the risk of distal stent-induced new entry (SINE) after stent insertion. The main stent used for this case is 34–26–160 mm with 8 mm tapering. The proximal and distal oversizing is 5.8% and 15.3%, respectively. The poststent oversizing ratio of cross-sectional area was also calculated, with the value of 0.553 and 0.811 for Post-1 and Post-2, respectively. Although the value of poststent oversizing ratio of area is not in the range of those with distal SINE (32, 33), either for Post-1 or Post-2. However, it increases from Post-1 to Post-2, which indicates a close follow-up is needed in the future. Furthermore, the change of tortuosity also shows that close monitor is necessary in the future to avoid the distal SINE (21).

Image-based CFD simulation has been widely accepted as an alternative approach to study the hemodynamic properties, due to the difficulty of studying the outcome directly *in vivo*. Hemodynamic indicators could help to evaluate the efficacy of treatment options, such as the Norwood procedure (34, 35), thoracic endovascular aortic repair and hybrid treatment procedure (13, 15). However, hemodynamic simulation might be limited for that focuses on the vessel wall motion or that aims to investigate the mechanical interaction between vessel wall and the stent grafts due to the simplification of rigid wall in hemodynamic analysis.

This study was conducted based on two longitudinal follow-up datasets of one patient who suffered from TBAD with ARSA. Indeed, large cohorts of patients and closed long-term follow-ups are warranted to draw more substantial clinical conclusions. However, the rare coexistence of TBAD and ARSA poses challenges for multiple case collections. In addition, boundary condition data of Post-2 came from the Doppler ultrasound data of Post-1 due to the absence of ultrasound measurement in Post-2. This might introduce deviations to the results, although boundary condition data calibration was conducted according to the geometrical features of the Post-2 model. Furthermore, to save computing time, flow analyses were based on CFD with the rigid wall assumption in the present study, which might overestimate the values of hemodynamic parameters. Although the simulation pipeline was previously validated by 4-dimensional phase-contrast magnetic resonance (36), accuracy needs to be continuously improved by performing fluid-structure interaction analysis, which could provide more detailed and accurate information on functional indictors in the future.

## Conclusions

In this study, a novel stent graft technique (EMSBSG) was applied to treat TBAD with ARSA and a hemodynamic assessment tool was proposed to evaluate the therapeutic effect for the aortic disease with complex morphology. It showed that EMSBSG could lead to the positive remodeling of the aorta and preserve the blood flow of aortic arch branches, confirming the efficacy of this technique in treating TBAD with ARSA. Hemodynamic indicators could also imply potential negative remodeling, thus informing the necessity of subsequent intervention.

## Data Availability Statement

The original contributions presented in the study are included in the article/Supplementary Material, further inquiries can be directed to the corresponding authors.

## Ethics Statement

The studies involving human participants were reviewed and approved by Chinese PLA General Hospital. The patients/participants provided their written informed consent to participate in this study.

## Author Contributions

DC, JZ, JX, and WG designed research studies. ZW and HD collected the patient data. XZ, MW, SL, SX, and HJ analyzed the data. DC, XZ, and JS wrote the manuscript. All authors contributed to the article and approved the submitted version.

## Funding

This study was supported by the Beijing Natural Science Foundation (Z190014, 7212094, and L192045), the National Natural Science Foundation of China (81970404 and 82170498), the Beijing Municipal Science and Technology Project (Z211100002921048), and the Jiangsu Health Commission Sponsored Medical Research Project (M2020007).

## Conflict of Interest

The authors declare that the research was conducted in the absence of any commercial or financial relationships that could be construed as a potential conflict of interest.

## Publisher's Note

All claims expressed in this article are solely those of the authors and do not necessarily represent those of their affiliated organizations, or those of the publisher, the editors and the reviewers. Any product that may be evaluated in this article, or claim that may be made by its manufacturer, is not guaranteed or endorsed by the publisher.

## Abbreviations

ARSA: aberrant right subclavian artery
TBAD: type B aortic dissection
EMSBSG: embedded modular single-branched stent graft
LSA: left subclavian artery
TL: true lumen
FL: false lumen
MAGR: main aortic grafting region
CFD: Computational fluid dynamics
WSS: wall shear stress
TAWSS: time-averaged wall shear stress
OSI: oscillatory shear index
RRT: relative residence time
LPD: luminal pressure difference
FBP: first balance position
AR: area ratio
SINE: stent-induced new entry.

## References

[1] Varetto G Castagno C Trevisan A Quaglino S Garneri P Mossetti C. Mediastinoscopy-assisted treatment of an aberrant right subclavian artery. Ann Vasc Surg. (2016) 31:210.e219–210.e211. 10.1016/j.avsg.2015.05.044 26631774

[2] Kikuchi K Makuuchi H Oono M Murakami H Suzuki T Ando T. Surgery for aortic dissection involving an aberrant right subclavian artery. Jpn J Thorac Cardiovasc Surg. (2005) 53:632–4. 10.1007/BF02665073 16408467

[3] Zhu J-M Qi R-D Liu Y-M Zheng J Xing X-Y Sun L-Z. Repair of complicated type B dissection with an aberrant right subclavian artery. Interact Cardiovasc Thorac Surg. (2016) 22:718–22. 10.1093/icvts/ivw043 26956707 PMC4986787

[4] Kieffer E Bahnini A Koskas F. Aberrant subclavian artery: surgical treatment in thirty-three adult patients. J Vasc Surg. (1994) 19:100–11. 10.1016/S0741-5214(94)70125-3 8301723

[5] Yang C Shu C Li M Li Q Kopp R. Aberrant subclavian artery pathologies and Kommerell's diverticulum: a review and analysis of published endovascular/hybrid treatment options. J Endovasc Ther. (2012) 19:373–82. 10.1583/11-3673MR.1 22788890

[6] Ding H Luo S Liu Y Huang W Jiang M Li J. Outcomes of hybrid procedure for type B aortic dissection with an aberrant right subclavian artery. J Vasc Surg. (2018) 67:704–11. 10.1016/j.jvs.2017.07.124 28993035

[7] Vos AWF Wisselink W Rijbroek A Avontuur JA Manoliu RA Rauwerda JA. Endovascular repair of a Type B aortic dissection with transposition of a coexistent aberrant subclavian (Lusorian) artery. J Endovasc Ther. (2002) 9:549–53. 10.1177/152660280200900427 12223019

[8] Dueppers P Reutersberg B Rancic Z Messmer F Menges A-L Meuli L. Long-term results of total endovascular repair of arch-involving aortic pathologies using parallel grafts for supra-aortic debranching. J Vasc Surg. (2021) 75:813–23.e1. 10.1016/j.jvs.2021.09.020 34606961

[9] Chen D Zhang X Mei Y Liao F Xu H Li Z. Multi-stage learning for segmentation of aortic dissections using a prior aortic anatomy simplification. Med Image Anal. (2021) 69:101931. 10.1016/j.media.2020.101931 33618153

[10] Mora H Mora-Pascual JM García-García A Martínez-González P. Computational analysis of distance operators for the iterative closest point algorithm. PLoS ONE. (2016) 11:e0164694. 10.1371/journal.pone.0164694 27768714 PMC5074461

[11] Grinberg L Karniadakis GE. Outflow boundary conditions for arterial networks with multiple outlets. Ann Biomed Eng. (2008) 36:1496–514. 10.1007/s10439-008-9527-7 18612828

[12] Li Z Liang S Xu H Zhu M Mei Y Xiong J. Flow analysis of aortic dissection: comparison of inflow boundary conditions for computational models based on 4D PCMRI and Doppler ultrasound. Comput Methods Biomech Biomed Eng. (2021) 24:1251–62. 10.1080/10255842.2021.1876036 33522843

[13] Xu H Li Z Dong H Zhang Y Wei J Watton PN. Hemodynamic parameters that may predict false-lumen growth in type-B aortic dissection after endovascular repair: a preliminary study on long-term multiple follow-ups. Med Eng Phys. (2017) 50:12–21. 10.1016/j.medengphy.2017.08.011 28890304

[14] Chen D Müller-Eschner M Kotelis D Böckler D Ventikos Y von Tengg-Kobligk H. longitudinal study of Type-B aortic dissection and endovascular repair scenarios: computational analyses. Med Eng Phys. (2013) 35:1321–30. 10.1016/j.medengphy.2013.02.006 23523079

[15] Xu H Xiong J Han X Mei Y Shi Y Wang D. Computed tomography-based hemodynamic index for aortic dissection. J Thorac Cardiovasc Surg. (2021) 162:e165–76. 10.1016/j.jtcvs.2020.02.034 32217023

[16] Itatani K Miyaji K Qian Y Liu JL Miyakoshi T Murakami A. Influence of surgical arch reconstruction methods on single ventricle workload in the Norwood procedure. J Thorac Cardiovasc Surg. (2012) 144:130–8. 10.1016/j.jtcvs.2011.08.013 21907359

[17] Numata S Itatani K Kanda K Doi K Yamazaki S Morimoto K. Blood flow analysis of the aortic arch using computational fluid dynamics †. Eur J Cardiothorac Surg. (2016) 49:1578–85. 10.1093/ejcts/ezv459 26792932

[18] Menichini C Cheng Z Gibbs RGJ Xu XY. Predicting false lumen thrombosis in patient-specific models of aortic dissection. J R Soc Interface. (2016) 13:20160759. 10.1098/rsif.2016.0759 27807275 PMC5134025

[19] Xu H Mei Y Han X Wei J Watton PN Jia W. Optimization schemes for endovascular repair with parallel technique based on hemodynamic analyses. Int J Numer Method Biomed Eng. (2019) 35:e3197. 10.1002/cnm.3197 30838798

[20] van Bakel TM Arthurs CJ van Herwaarden JA Moll FL Eagle KA Patel HJ. A computational analysis of different endograft designs for Zone 0 aortic arch repair†. Eur J Cardiothorac Surg. (2018) 54:389–96. 10.1093/ejcts/ezy068 29554234

[21] Dong Z Fu W Wang Y Wang C Yan Z Guo D. Stent graft-induced new entry after endovascular repair for Stanford type B aortic dissection. J Vasc Surg. (2010) 52:1450–7. 10.1016/j.jvs.2010.05.121 20800417

[22] Dobson G Flewitt J Tyberg JV Moore R Karamanoglu M. Endografting of the descending thoracic aorta increases ascending aortic input impedance and attenuates pressure transmission in dogs. Eur J Vasc Endovasc Surg. (2006) 32:129–35. 10.1016/j.ejvs.2006.01.020 16564712

[23] Tzilalis VD Kamvysis D Panagou P Kaskarelis I Lazarides MK Perdikides T. Increased pulse wave velocity and arterial hypertension in young patients with thoracic aortic endografts. Ann Vasc Surg. (2012) 26:462–7. 10.1016/j.avsg.2011.06.021 22284778

[24] Lantelme P Dzudie A Milon H Bricca G Legedz L Chevalier J-M. Effect of abdominal aortic grafts on aortic stiffness and central hemodynamics. J Hypertens. (2009) 27:1268–76. 10.1097/HJH.0b013e3283299b22 19342960

[25] Benetos A Safar M Rudnichi A Smulyan H Richard J-L Ducimetière P. Pulse pressure. Hypertension. (1997) 30:1410–5. 10.1161/01.HYP.30.6.1410 9403561

[26] Takeda Y Sakata Y Ohtani T Tamaki S Omori Y Tsukamoto Y. Endovascular aortic repair increases vascular stiffness and alters cardiac structure and function. Circ J. (2013) 78:322–8. 10.1253/circj.CJ-13-0877 24292128

[27] Polanczyk A Piechota-Polanczyk A Stefańczyk L Strzelecki M. Spatial configuration of abdominal aortic aneurysm analysis as a useful tool for the estimation of stent-graft migration. Diagnostics. (2020) 10:737. 10.3390/diagnostics10100737 32977588 PMC7598279

[28] Li Z Kleinstreuer C. Analysis of biomechanical factors affecting stent-graft migration in an abdominal aortic aneurysm model. J Biomech. (2006) 39:2264–73. 10.1016/j.jbiomech.2005.07.010 16153654

[29] Rayz VL Boussel L Ge L Leach JR Martin AJ Lawton MT. Flow residence time and regions of intraluminal thrombus deposition in intracranial aneurysms. Ann Biomed Eng. (2010) 38:3058–69. 10.1007/s10439-010-0065-8 20499185 PMC2940011

[30] Wu MH-D Kouchi Y Onuki Y Shi Q Yoshida H Kaplan S. Effect of differential shear stress on platelet aggregation, surface thrombosis, and endothelialization of bilateral carotid-femoral grafts in the dog. J Vasc Surg. (1995) 22:382–92. 10.1016/S0741-5214(95)70005-6 7563399

[31] Chen D Liang S Li Z Mei Y Dong H Ma Y. A Mock Circulation loop for *in vitro* hemodynamic evaluation of aorta: application in aortic dissection. J Endovasc Ther. (2021) 29:132–42. 10.1177/15266028211034863 34342237

[32] Pantaleo A Jafrancesco G Buia F Leone A Lovato L Russo V. Distal stent graft-induced new entry: an emerging complication of endovascular treatment in aortic dissection. Ann Thorac Surg. (2016) 102:527–32. 10.1016/j.athoracsur.2016.02.001 27112653

[33] Weng S-H Weng C-F Chen W-Y Huang C-Y Chen IM Chen C-K. Reintervention for distal stent graft-induced new entry after endovascular repair with a stainless steel-based device in aortic dissection. J Vasc Surg. (2013) 57:64–71. 10.1016/j.jvs.2012.07.006 23141675

[34] Qian Y Liu JL Itatani K Miyaji K Umezu M. Computational hemodynamic analysis in congenital heart disease: simulation of the norwood procedure. Ann Biomed Eng. (2010) 38:2302–13. 10.1007/s10439-010-9978-5 20195758

[35] Wen J Yuan D Wang Q Hu Y Zhao J Zheng T. A computational simulation of the effect of hybrid treatment for thoracoabdominal aortic aneurysm on the hemodynamics of abdominal aorta. Sci Rep. (2016) 6:23801. 10.1038/srep23801 27029949 PMC4814838

[36] Pirola S Guo B Menichini C Saitta S Fu W Dong Z. 4-D Flow MRI-based computational analysis of blood flow in patient-specific aortic dissection. IEEE Trans Biomed Eng. (2019) 66:3411–9. 10.1109/TBME.2019.2904885 30872222

