# Supplementary material for: Functional Evaluation of Embedded Modular Single-Branched Stent Graft: Application to Type B Aortic Dissection With Aberrant Right Subclavian Artery
Source: Front Cardiovasc Med. 2022 May 2;9:869505. doi: 10.3389/fcvm.2022.869505 (PMC9108238; doi:10.3389/fcvm.2022.869505)
Supplement: Supplementary file 1 [file Data_Sheet_1.docx]

Supplementary Material

**S1. Details to perform the** **endovascular grafting procedure**

Implantation was performed under general anesthesia in a hybrid operating room. Bilateral brachial arteries and bilateral femoral arteries were punctured, and systemic heparinization was achieved with intravenous bolus injection of 100 IU/kg of unfractionated heparin. Endovascular repair involved the following steps:

- Through the left femoral approach, a 13*50mm Viabahn covered stent graft (SG) was aligned with the orifice of left subclavian artery (LSA) and then deployed into LSA.
- According to the measurement, a 34*26*160mm embedded modular single-branched stent graft (EMSBSG) (WFT-TE-3426160-1040) was selected. The pre-guidewire of the EMSBSG was delivered from right femoral artery and through out from right brachial artery. Thereafter, the delivery system was introduced through right femoral artery access. Deployed the EMSBSG until the proximal end of EMSBSG was flush with the distal edge of LSA and the proximal edge of graft was aligned with the orifice of aberrant right subclavian artery (ARSA).
- Through the guidewire in ARSA, an 11*50mm branched SG (WFT-TL-1111050-L) was deployed in ARSA with a full overlap with the embedded branched port in the aortic main body SG.
- An 11*40mm branched SG (WFT-TL-1111040-L) was connected to the branched SG in ARSA with an overlap of at least 2cm. Thereafter, a 10mm balloon was used to expand the junction of the two branched SGs.

Completion angiography revealed exclusion of the dissection and patency of LSA, ARSA, and bilateral vertebral arteries without endoleak. The patient was discharged on postoperative day 5 without complications.

**S2. Imaging acquisitions via CTA scans**

The patient underwent three CT-angiography (CTA) scans before operation (Pre-1) and at 4 and 25 days after EMSBSG implantation (Post-1 and Post-2). All three CTA datasets were acquired via a dual‐source CT scanner (Brilliance iCT256, Royal Philips, Dutch). The CTAs of the aorta were carried out with injection of 70~90ml of contrast with 50ml of saline chaser, threshold 80HU; rotation speed: 500ms; collimation: 64; pitch: 1.0; voltage: 100kV; current: 200~350mA. DICOM files were exported and were prepared for image segmentation and three-dimensional (3D) vessel configuration reconstruction.

**S3. Calculation of morphological parameters**

Based on the 3D reconstructed aortic models, presumed stented regions were selected, true lumen (TL) centerline was subsequently calculated. A series of slices perpendicular to the TL centerline with an interval of 1.0 mm were extracted, as shown in Figure S1A.

Several morphological parameters, including area, circumference, equivalent diameter, transverse diameter, longitudinal diameter, and the ratio between the transverse and longitudinal diameter (aspect ratio), were computed from each of the selected slices in the reconstructed aortic models. In detail, a few lines of intersection were generated when the perpendicular slice of each centerline point intersected with the 3D model, which should be fine enough to show the basic geometric characteristics of the boundary of each extracted slice. The circumference of each slice was the total length of the lines of intersection and the area of each slice was the sum area of triangles composed of the corresponding centerline point and the lines of intersection, as shown in the Figure S1B. Equivalent diameter of each slice was defined as the diameter of the circle, whose area was the same as the slice, as shown in Eq.S1.


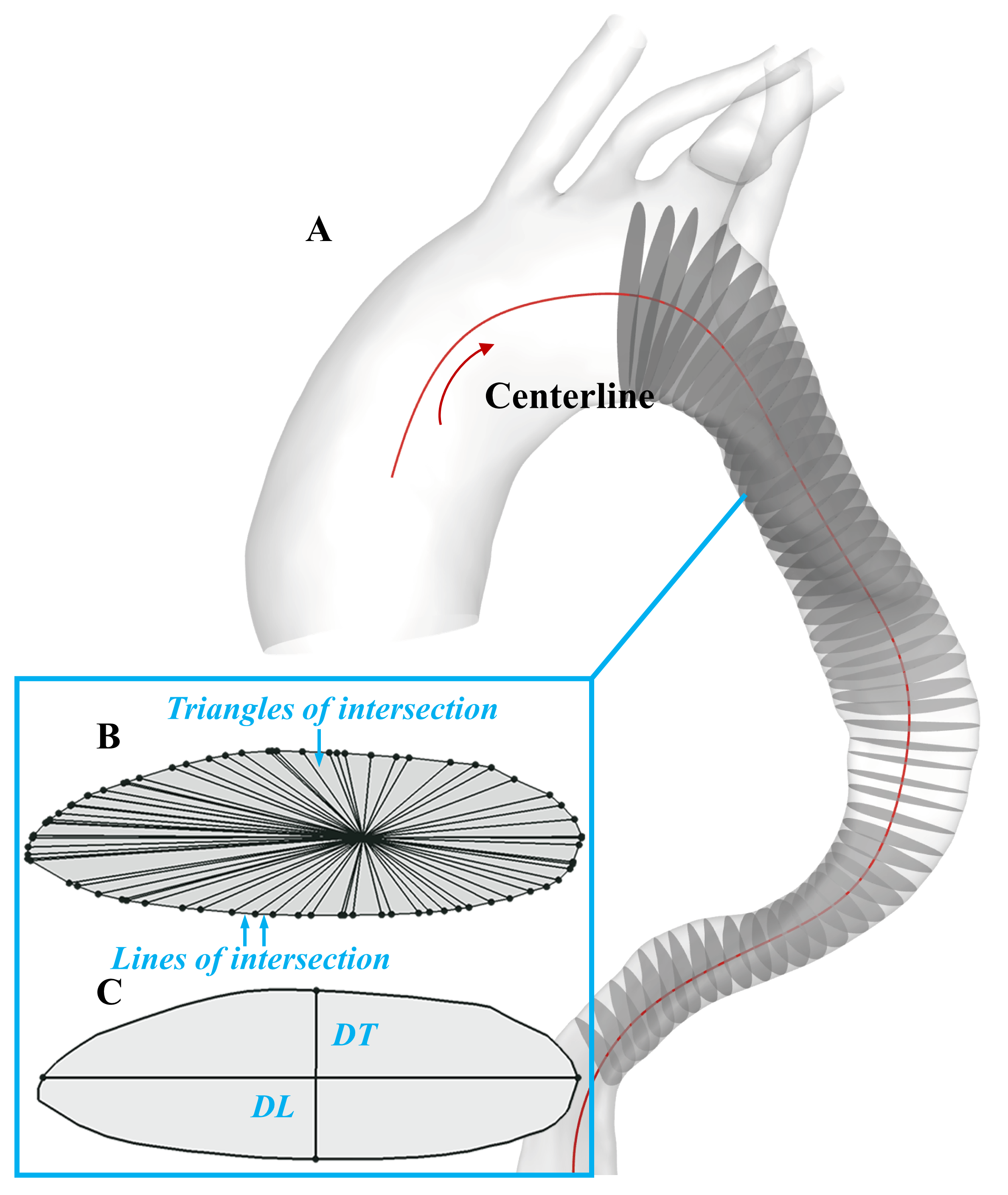


Figure S1 Measurements of morphological parameters (A) shows slice extraction of a representative case. (B) displays measurement of circumference and area. (C) illustrates measurement of orthogonal diameter. *DT* = Transverse diameter; *DL* = Longitudinal diameter

$\text{Equivalent Diameter=2*}\sqrt{\frac{\text{Area}}{\pi}}$ Eq.S1

Orthogonal diameter of each slice is calculated to acquire transverse diameter, longitudinal diameter and aspect ratio, as shown in the Figure S1C. Automatic procedures of parameter measurements based on the 3D aortic models were achieved by an in-house program running in MATLAB (R2019b, The Mathworks, Natick, MA).

**S4. Doppler Ultrasound Measurements and Velocity Boundary Conditions**

Table S1 shows the detailed parameters of the measurement. The velocity of ascending aorta (AAo) was measured through the apical 5-chamber view and the suprasternal long axis view of aortic arch. The two results have been compared to each other to ensure the maximum velocity at AAo could be captured. On the other hand, the velocity at other sites was measured at the proximal and distal region of the specific vessel (detailed positions refer to Table S1). The two results of one particular vessel have been compared. If the difference is more than 5%, the measurement should be re-done, in order to ensure the accuracy of the measured velocity. At each measurement site, appropriate ultrasound probe was employed (Table S1), the Doppler gate was positioned at the center of the blood vessel, and the Doppler angle cursor was accurately aligned with the vessel axis. Figure S2 shows the velocity boundary conditions for Pre-1, Post-1 and Post-2.

Table S1. Parameters of Doppler ultrasound velocimetry

| Site | Position | | View | Doppler Angle |
| --- | --- | --- | --- | --- |
| Ascending aorta | - | 2.5cm above aortic valve | Suprasternal long axis view of aortic arch | 30 |
|  |  |  | apical 5-chamber view | 22 |
| Right common carotid artery | Distal | 0.8cm below bifurcation | long axis view | 47 |
|  | Proximal | 0.9cm above aortic arch |  | 47 |
| Left common carotid artery | Distal | 3.0cm below bifurcation | long axis view | 47 |
|  | Proximal | 1.1cm above aortic arch |  | 36 |
| Left subclavian artery | Distal | 2.8cm above aortic arch | long axis view | 30 |
|  | Proximal | 1.0cm above aortic arch |  | 36 |
| Aberrant right subclavian artery | Distal | 3.2cm above aortic arch | long axis view | 35 |
|  | Proximal | 1.0cm above aortic arch |  | 30 |

**S5. Hemodynamic computations**


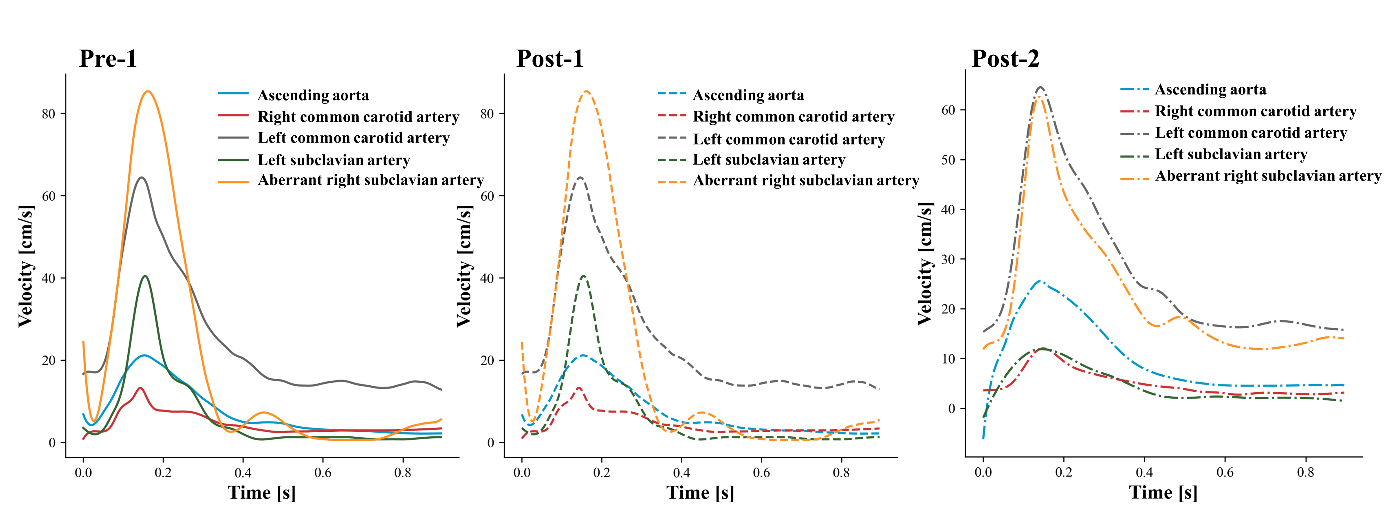


Figure S2 displays the velocity boundary conditions for Pre-1, Post-1 and Post-2.

The general model of fluid momentum is given by the Navier-Stokes equation (Eq.S2), where *ρ* is the fluid density, ***u*** is the flow velocity vector, *t* is time, ∇ is the gradient operator, *p* is the hydrodynamic pressure, [τ] is the stress tensor, and ***F****_b_* represents the body forces. In this study, blood is modeled as Newtonian, incompressible and isothermal fluid without body force, with constant viscosity *μ* and constant density *ρ*. The simplification of the general momentum equation then reads as Eq.S3, where Δ is the Laplacian operator. Considering there are no sources of blood inside the aorta, the flow field also satisfy mass conservation, which is expressed by the continuity equation (Eq.S4). The Eq.S3 and S4 were then solved by the Finite Volume Method in CFD-ACE (ESI Group, Paris, France). A second order accurate discretization (central differences) was used to solve the flow velocity. Algebraic MultiGrid acceleration was employed and the SIMPLEC-type pressure correction was used for pressure–velocity coupling.

$\rho\left(\frac{\partial u}{\partial t}+u\cdot\nabla u\right)=F_{b}-\nabla p-\nabla\cdot[\tau]$ Eq.S2

$\rho\left(\frac{\partial u}{\partial t}+u\cdot\nabla u\right)=-\nabla p+\mu\Delta u$ Eq.S3

$\nabla\cdot u=0$ Eq.S

**S6. WSS-based parameter calculation**

MATLAB (R2019b, The Mathworks, Natick, MA) was employed for post processing which allow averaging of the parameters over a specified period of time. The average wall shear stress (WSS) of each wall cell throughout a cardiac cycle was evaluated via the time-averaged wall shear stress (TAWSS), which is obtained from Eq.S5.

 Eq.S5

Where *t* and *c* are the cardiac cycle period and cell on the arterial wall, respectively. The cyclic variation of WSS is often expressed using oscillatory shear index (OSI), which is intended to show flow that contains azimuthal variation in direction (1) and is defined as Eq.S6. OSI has a range between 0 a maximum of 0.5, where 0.5 indicates purely oscillatory flow.

 Eq.S6

Relative residence time (RRT) refers to the relative time for a particle stagnating in an area(2) and is defined as the combination of TAWSS and OSI, given by Eq.S7.

 Eq.S7

**S7. Tear information at different time points**

Table S2. The tear information of the studied patient

| Time-Points | Number of tears^1*^ | Location of the tears^**^ (mm) | | | Area of tear  (mm^2^) |
| --- | --- | --- | --- | --- | --- |
|  |  | Type of  Tear^1^ | Linear distance | Curve distance |  |
| Pre-1 | 4 | Primary tear | 55.24 | 61.38 | 152.93 |
|  |  | Re-entry #1 | 17.74 | 17.92 | 96.21 |
|  |  | Re-entry #2 | 191.74 | 355.07 | 10.35 |
|  |  | Re-entry #3 | 240.14 | 428.83 | 37.25 |
| Post-1 | 2 | Re-entry #1 | 191.74 | 355.07 | 7.20 |
|  |  | Re-entry #2 | 240.14 | 428.83 | 35.72 |
| Post-2 | 2 | Re-entry #1 | 191.74 | 355.07 | 7.00 |
|  |  | Re-entry #2 | 240.14 | 428.83 | 18.87 |

^*^ The tears along the aorta were counted and measured, while those in the iliac arteries were not included.

^**^ The centerline of the true lumen was extracted for each geometric model. The highest position which was presented in the aortic arch region was assigned as the reference point for each model, and the straight-line distance and the curve distance along the centerline between this reference point to the centroid of each tear were measured, regarded as the location of the tears.

^1^ The positions of re-entry tears (Re-entry #2 and #3) did not change after stent graft implantation.

**S8. Flow and pressure patterns of other stent techniques**

Two cases with different stent techniques were randomly selected from our database and computed. One case (Case #1) was treated with provisional extension to induce complete attachment (PETTICAOT) technique (with bare mental stent), the other (Case #2) was handled with traditional TEVAR technique (without bare mental stent).


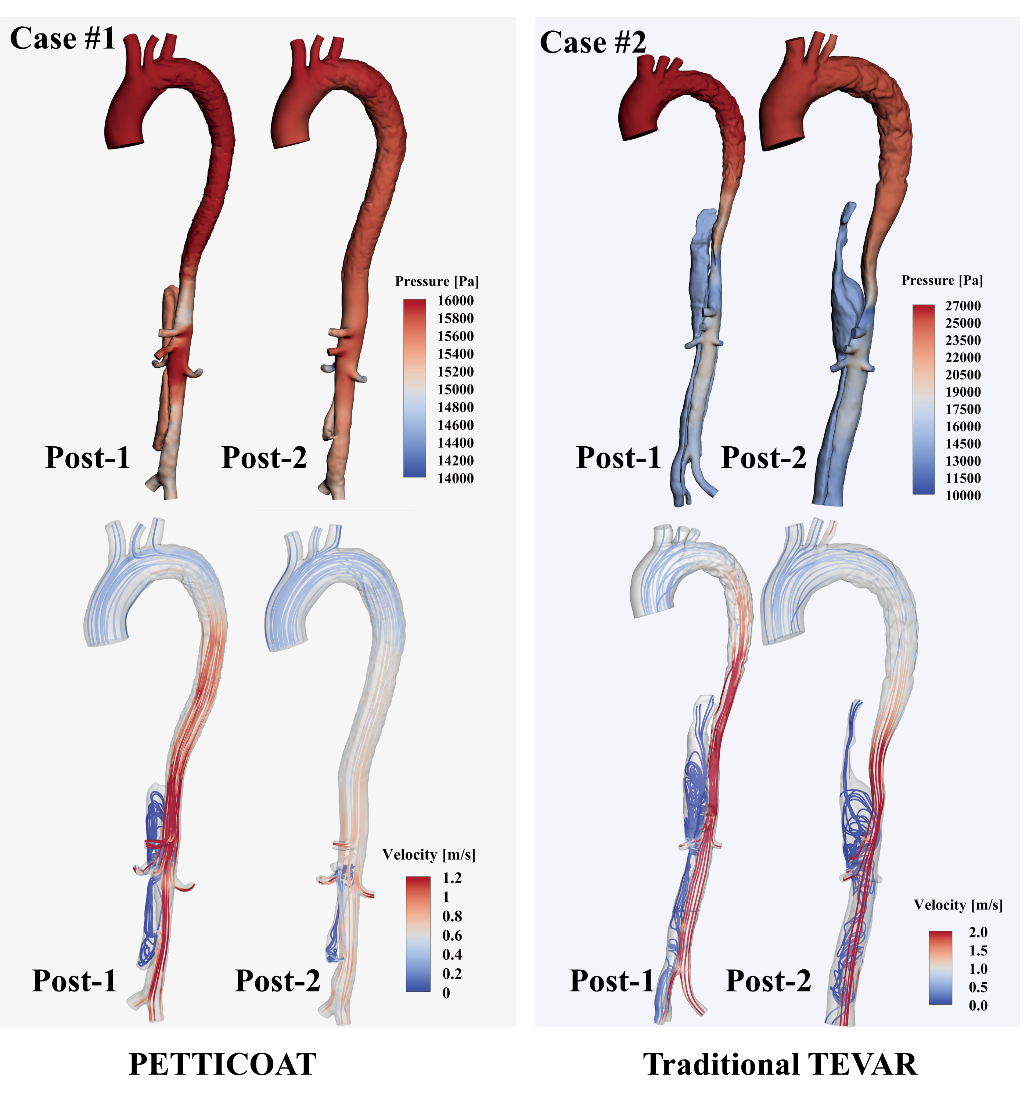


Figure S3 Figure S2 Pressure and flow patterns of cases with other stent techniques. Case #1 experienced PETTICOAT. Case #2 received traditional TEVAR treatment. PETTICOAT = Provisional extension to induce complete attachment; TEVAR = Thoracic endovascular aortic repair.

**S9. Analysis of** **abdominal region**

To investigate the prognosis in the abdominal region after EMSBSG insertion, TAWSS and RRT in the whole dissected region for Post-1 and Post-2 were displayed, as show in Figure S4. The re-entry tears that located in the abdominal region in Post-2 was also displayed to support the analysis, as shown in Figure S5.


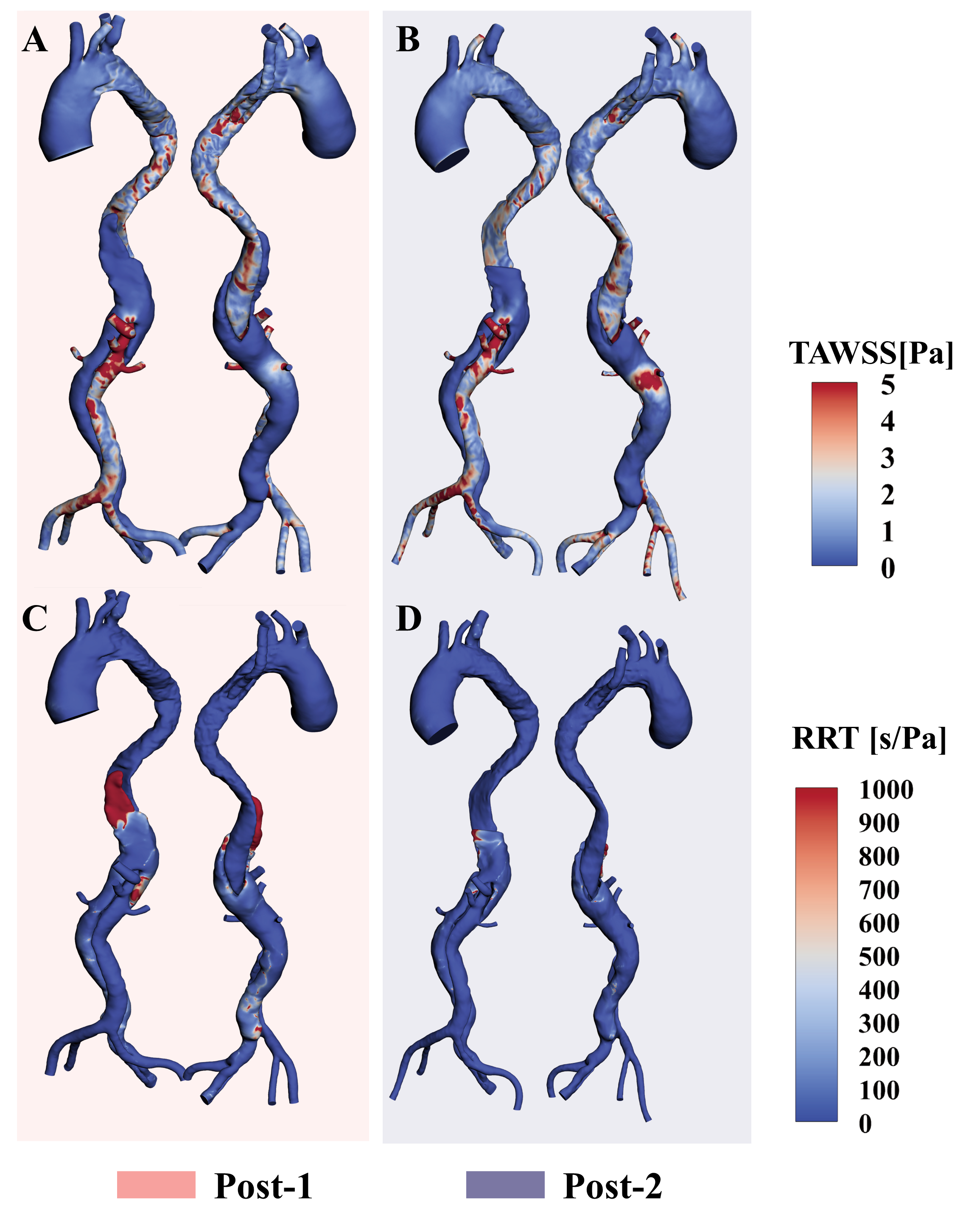


Figure S4 (A) – (B) show the TAWSS distributions for Post-1 and Post-2, respectively. (C) – (D) display the results of RRT contour plots for Post-1 and Post-2, respectively. TAWSS = Time-averaged wall shear stress; RRT = Relative residence time.


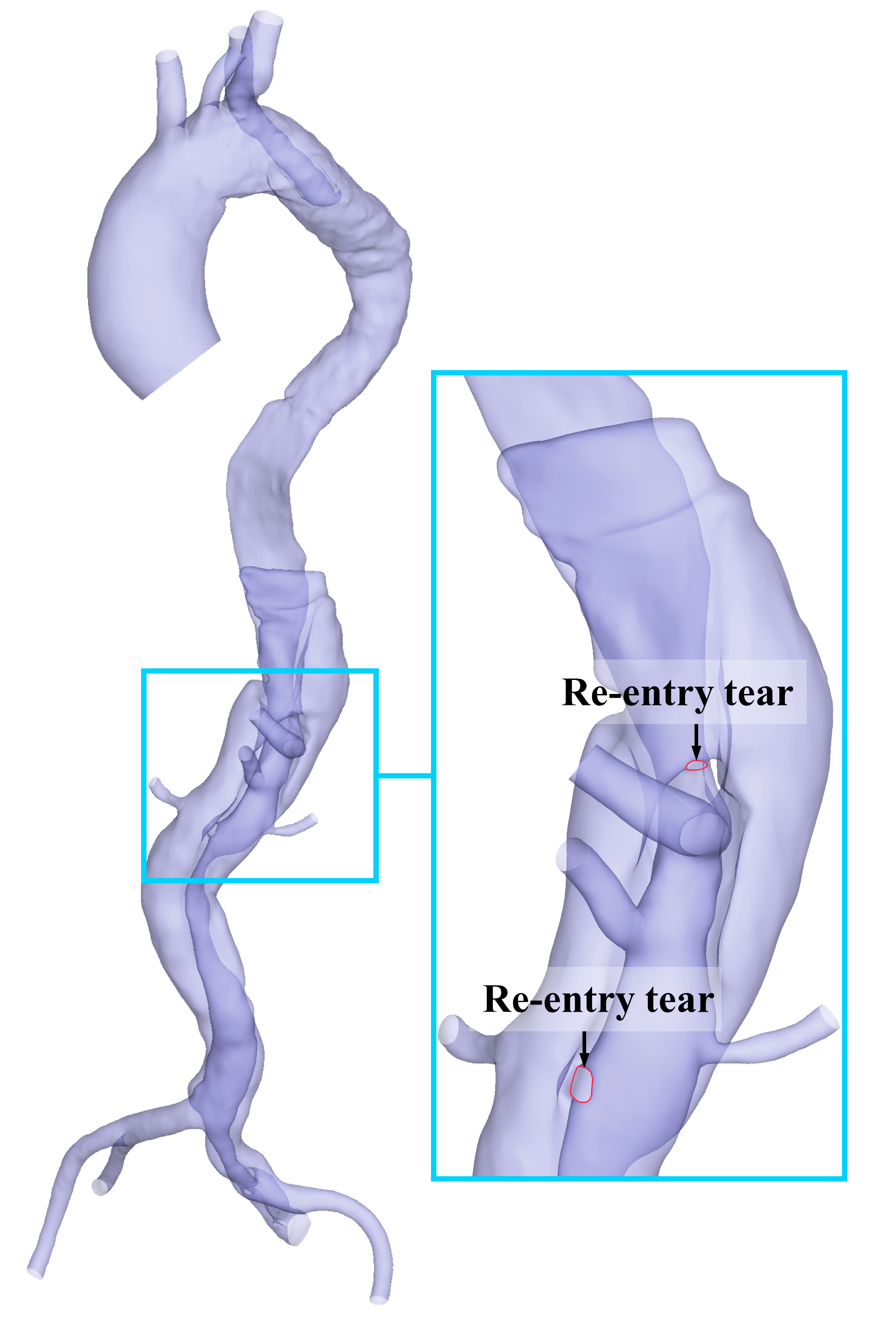


Figure S5 shows the positions of re-entry tears locating in the abdominal region in Post-2.

**Supplementary References**

1. Sotelo J, Urbina J, Valverde I, Tejos C, Irarrázaval P, Andia ME, et al. 3D Quantification of Wall Shear Stress and Oscillatory Shear Index Using a Finite-Element Method in 3D CINE PC-MRI Data of the Thoracic Aorta. IEEE Trans Med Imaging. 2016;35:1475-1487.doi:10.1109/TMI.2016.2517406

2. Papadopoulos KP, Gavaises M, Pantos I, Katritsis DG, Mitroglou N. Derivation of flow related risk indices for stenosed left anterior descending coronary arteries with the use of computer simulations. Med Eng Phys. 2016;38:929-939.doi:<https://doi.org/10.1016/j.medengphy.2016.05.016>
